# Identification of cuproptosis-related subtypes and the development of a prognostic model in glioma

**DOI:** 10.3389/fgene.2023.1124439

**Published:** 2023-03-01

**Authors:** Zhaoping Wu, Wei Li, Hecheng Zhu, Xuewen Li, Yi Zhou, Quan Chen, Haoxuan Huang, Wenlong Zhang, Xingjun Jiang, Caiping Ren

**Affiliations:** ^1^ Department of Neurosurgery, National Clinical Research Center for Geriatric Disorders, Xiangya Hospital, Central South University, Changsha, Hunan, China; ^2^ Department of Clinical Pharmacology, Hunan Key Laboratory of Pharmacogenetics and National Clinical Research Center for Geriatric Disorders, Xiangya Hospital, Central South University, Changsha, China; ^3^ Institute of Clinical Pharmacology, Central South University, Changsha, China; ^4^ Changsha Kexin Cancer Hospital, Changsha, Hunan, China; ^5^ Cancer Research Institute, School of Basic Medical Science, Central South University, Changsha, China; ^6^ The NHC Key Laboratory of Carcinogenesis and The Key Laboratory of Carcinogenesis and Cancer Invasion of the Chinese Ministry of Education, Central South University, Changsha, China

**Keywords:** cuproptosis, prognostic model, gene subtypes, tumor microenvironment, glioma

## Abstract

**Introduction:** A copper-dependent cell death, cuproptosis, involves copper binding with lipoylated tricarboxylic acid (TCA) cycle components. In cuproptosis, ferredoxin 1 (FDX1) and lipoylation act as key regulators. The mechanism of cuproptosis differs from the current knowledge of cell death, which may invigorate investigations into copper’s potential as a cancer treatment. An extremely dismal prognosis is associated with gliomas, the most prevalent primary intracranial tumor. In patients with glioma, conventional therapies, such as surgery and chemotherapy, have shown limited improvement. A variety of cell death modes have been confirmed to be operative in glioma oncogenesis and participate in the tumor microenvironment (TME), implicated in glioma development and progression. In this study, we aimed to explore whether cuproptosis influences glioma oncogenesis.

**Methods:** Gene expression profiles related to cuproptosis were comprehensively evaluated by comparing adjacent tissues from glioma tissues in The Cancer Genome Atlas (TCGA) (https://portal.gdc.cancer.gov/) database. Gene expression, prognostic, clinical, and pathological data of lower-grade gliomas (LGG) and glioblastoma were retrieved from TCGA and Gene Expression Omnibus (GEO) (https://www.ncbi.nlm.nih.gov/geo/) databases. The datasets were managed by “Combat” algorithm to eliminate batch effects and then combined. A consensus clustering algorithm based on the Partitioning Around Medoid (PAM) algorithm was used to classified 725 patients with LGG and glioblastoma multiforme (GBM) into two cuproptosis subtypes. According to the differentially expressed genes in the two cuproptosis subtypes, 725 patients were divided into 2 gene subtypes. Additionally, a scoring system that associated with TME was constructed to predict patient survival and patient immunotherapy outcomes. Furthermore, we constructed a prognostic CRG-score and nomogram system to predict the prognosis of glioma patients. 95 tissue specimens from 83 glioma patients undergoing surgical treatment were collected, including adjacent tissues. Using immunohistochemistry and RT-qPCR, we verified cuproptosis-related genes expression and CRG-score predictive ability in these clinical samples.

**Results:** Our results revealed extensive regulatory mechanisms of cuproptosis-related genes in the cell cycle, TME, clinicopathological characteristics, and prognosis of glioma. We also developed a prognostic model based on cuproptosis. Through the verifications of database and clinical samples, we believe that cuproptosis affects the prognosis of glioma and potentially provides novel glioma research approaches.

**Conclusion:** We suggest that cuproptosis has potential importance in treating gliomas and could be utilized in new glioma research efforts.

## 1 Introduction

Cuproptosis is a type of copper-dependent cell death that occurs by the binding of copper to the lipoylated components of the tricarboxylic acid (TCA) cycle. Lipoylated protein aggregation and subsequent loss of iron-sulfur cluster proteins cause cell death (Tang et al., 2022; Tsvetkov et al., 2022; Wang et al., 2022). Cuproptosis differs from the currently known mechanism of cell death and may invigorate research into copper’s potential as a cancer treatment (Kahlson and Dixon, 2022). Ferredoxin 1 (*FDX1*) and lipoylation are considered key regulators of cuproptosis. Six genes related to the lipoic acid pathway (*LIPT1*, *LIAS*, and *DLD*) and protein targets of lipoylation (*DLAT*, *PDHA1*, and *PDHB*) (Solmonson and DeBerardinis, 2018; Tang et al., 2022; Wang et al., 2022) were thought to be positive regulatory genes. In addition, pyruvate dehydrogenase complex-related genes, including *MTFS*, *GLS*, and *CDKN2A*, are considered negative regulators (Tsvetkov et al., 2022). Protein lipoylation is known to occur on only four enzymes: *DBT*, *GCSH*, *DLST*, and *DLAT* (Rowland et al., 2018; Solmonson and DeBerardinis, 2018). It has been found that knocking out either *FDX1* or lipoylation-related enzymes can relieve copper toxicity in cells. The copper importer *SLC31A1 (CTR1)*, and exporters *ATP7A* (Aubert et al., 2020) and *ATP7B* are related to the homeostatic mechanisms that maintain intracellular copper concentrations. *SLC31A1* plays a key role in high-affinity Cu uptake (Lutsenko, 2010; Tang et al., 2022). *ATP7A* and *ATP7B* are closely related to Cu-transportation (Nevitt et al., 2012; Polishchuk et al., 2019). *NFE2L2* and *NL RP3* affect copper metabolism in hepatocellular carcinoma and Wilson’s disease (Dong et al., 2021; Ren et al., 2021). Thus, we had reason to believe that the 19 genes mentioned above may be cuproptosis-related genes and made them the basis of this study.

Glioma has a poor prognosis, and is the most prevalent primary intracranial tumor (Ostrom et al., 2014). Glioma patients have had limited prognosis improvement with conventional treatment options, including surgery and chemotherapy (Xu et al., 2020). Gliomas are classified into 4 grades in the 2021 World Health Organization (WHO) classification of central nervous system (CNS) tumors, with grades 1 and 2 being low-grade gliomas and grades 3 and 4 being high-grade gliomas (Louis et al., 2021). However, grades 2 and 3 gliomas are named lower-grade gliomas (LGG) according to the principles of common databases. In this study, we used naming rules of databases and classified LGG as grade 2 and 3 glioma, HGG as grade 4 glioma. A variety of cell death modes have been confirmed to be present in glioma and participate in the tumor microenvironment (TME) (Cai et al., 2022; Liu et al., 2022). Cuproptosis may play a role in glioma development and progression of glioma.

In this study, we aimed to explore whether cuproptosis influences glioma prognosis. We comprehensively evaluated the expression profiles of cuproptosis-related genes. Using cuproptosis-related genes from a survey of the literature, we were able to classify 725 patients with LGG and glioblastoma multiforme (GBM) into two subtypes. And then they were divided into 2 gene subtypes based on differentially expressed genes. Finally, our scoring system predicts patient survival and identifies the TME landscape of gliomas, enabling us to predict the treatment outcome of patients.

## 2 Materials and methods

### 2.1 Data sources

Gene expression, prognostic, clinical, and pathological data of gliomas were downloaded from The Cancer Genome Atlas (TCGA) (gene expression: 701 glioma and 5 adjacent samples; clinical: 1,104 samples; mutation: 984 samples) and Gene Expression Omnibus (GEO) (50 glioma samples) databases. The datasets were managed by “Combat” algorithm to eliminate batch effects and then combined.

### 2.2 Construction and analysis of cuproptosis subtypes

A consensus clustering algorithm based on Partitioning Around Medoid (PAM) algorithm was be used to categorize patients into cuproptosis subtypes. An analysis of patients’ clinicopathological characteristics and prognosis was carried out in order to determine the clinical features of the two subtypes. Cohort information included age, sex, glioma grade, and cuproptosis-related gene expression. We plotted the Kaplan-Meier curve with “survival” and “survminer” packages. Using “pheatmap” package, we generated a heatmap which would intuitively show the patient characteristics. GSVA and ssGSEA analyses revealed the TME differences between the two subtypes. ESTIMATE algorithm was used to calculate the immune and stromal scores of the cohort patients.

### 2.3 Differentially expressed genes identification and analysis

We used “limma” package to analyze differentially expressed genes between the two cuproptosis subtypes, with a standard of logFCfiter = 0.585 and adjusted *p*-value fliter = 0.05. To determine the potential functions of these genes, we used “clusterProfiler” and “enrichplot” to perform functional annotation of GO and analysis of KEGG pathway enrichment.

### 2.4 Construction of the cuproptosis-related genes score (CRG-score)

“Caret” package was used to randomly assigned the cohort patients to a training and test group (ratio of 1:1). We then analyzed the differentially expressed genes using LASSO and multivariate Cox analyses to select prognostic genes related to the gene subtypes. “Glmnet” package was used to avoid over-fitting.

$$CRG-score=\sum i\left(coef\;i\;*\;gene\;i\;expression\right)$$

We calculated the CRG-score for each patient. Patients were divided into a low- and high-risk group based on the median value of CRG-score.

### 2.5 Validation of the prognostic CRG-score

The predictive capacity of the CRG-score was verified with multiple R packages. We used “ggpubr” package to identify the risk score difference between cuproptosis subtypes and gene subtypes. Survival analysis was performed by “survival” and “survminer” packages. The receiver operating characteristic (ROC) analysis was based on “timeROC” package. We also used the “pheatmap” package to reveal CRG-score model genes expression in the low- and high-risk group. Sankey diagram was based on “ggplot2” and “ggalluvial” packages, which showed an entire network of the subtypes, risk of CRG-score, and survival status.

### 2.6 TME, mutation and cancer stem cell analysis

Using ESTIMATE algorithm, stromal and immune scores of the low- and high-risk groups were evaluated. Additionally, we used CIBERSORT algorithm to calculate human immune-cell subsets of each patient in the two risk groups. To analyze mutation and tumor mutation burden (TMB), we used “maftools” and “ggpubr” packages. Curve of cancer stem cell analysis was made by “ggpubr,” “ggplot2” and “ggExtra” packages.

### 2.7 Establishment of a cuproptosis-related nomogram system

A nomogram was established by the “rms,” “regplot” and “survival” packages. The patients’ age, sex, glioma grade, and risk cluster were given a specific score, and the total score of each patient was the sum of these various factor scores. Based on a patient’s total score, the nomogram predicted 1-, 3- and 5-year survival accurately, which has been evaluated by calibration plots.

### 2.8 Clinical tissue samples

Ninety five glioma tissue samples were donated by 83 patients who had undergone surgery at Xiangya Hospital, including 27 grade 2, 6 grade 3, 50 grade 4, and 12 adjacent tissues. This study was approved by the Ethics Committee of Xiangya Hospital of Central South University.

### 2.9 RT-qPCR and immunohistochemistry (IHC)

TRIzol (Invitrogen, 15596018) was used to extract RNA from glioma tissue after grinding in liquid nitrogen. Then we converted RNA to cDNA by the RevertAid RT Reverse Transcription Kit (Thermo Scientific, K1691). The RT-qPCR data were analysed by the 2^−ΔΔCT^ strategy and values were relative to the housekeeping gene Glyceraldehyde-3-Phosphate Dehydrogenase (*GAPDH*). A complete list of the primers used is provided in in Supplementary Table S1. The 95 glioma tissue samples were manufactured using a tissue microarray. The following antibodies were used for IHC: FDX1 (Proteintech, 12592-1-AP), PDHA1 (ABclonal, A1895), and DLST (ABclonal, A13297). The grading rules as follows: The intensity was scored as follows: 0, negative; 1, weak; 2, moderate; and 3, strong. The frequency of positive cells was defined as follows: 0, less than5%; 1, 5%–25%; 2, 26%–50%; 3, 51%–75%; and 4, greater than 75%. The final grade of staining was determined by multiplying the score for staining intensity with the score for the frequency of positive cells (values, 0–12). This grading rules can reduce the grading bias caused by different cell density.

### 2.10 Statistical analyses

We used Student’s t-test, Log-rank test, Cox regression model, GSVA analysis, ssGSEA analysis, PCA analysis, Least absolute shrinkage and selection operator (LASSO), multivariate Cox analysis, Spearman test and Wilcoxon test. All the statistical analyses were based on R (version 4.1.0). And the cutoffs for the Kaplan-meiers in this study was found by using the R package “survminer”.

## 3 Result

### 3.1 Genetic and transcriptional alterations of cuproptosis-related genes in glioma

**FIGURE 1:**
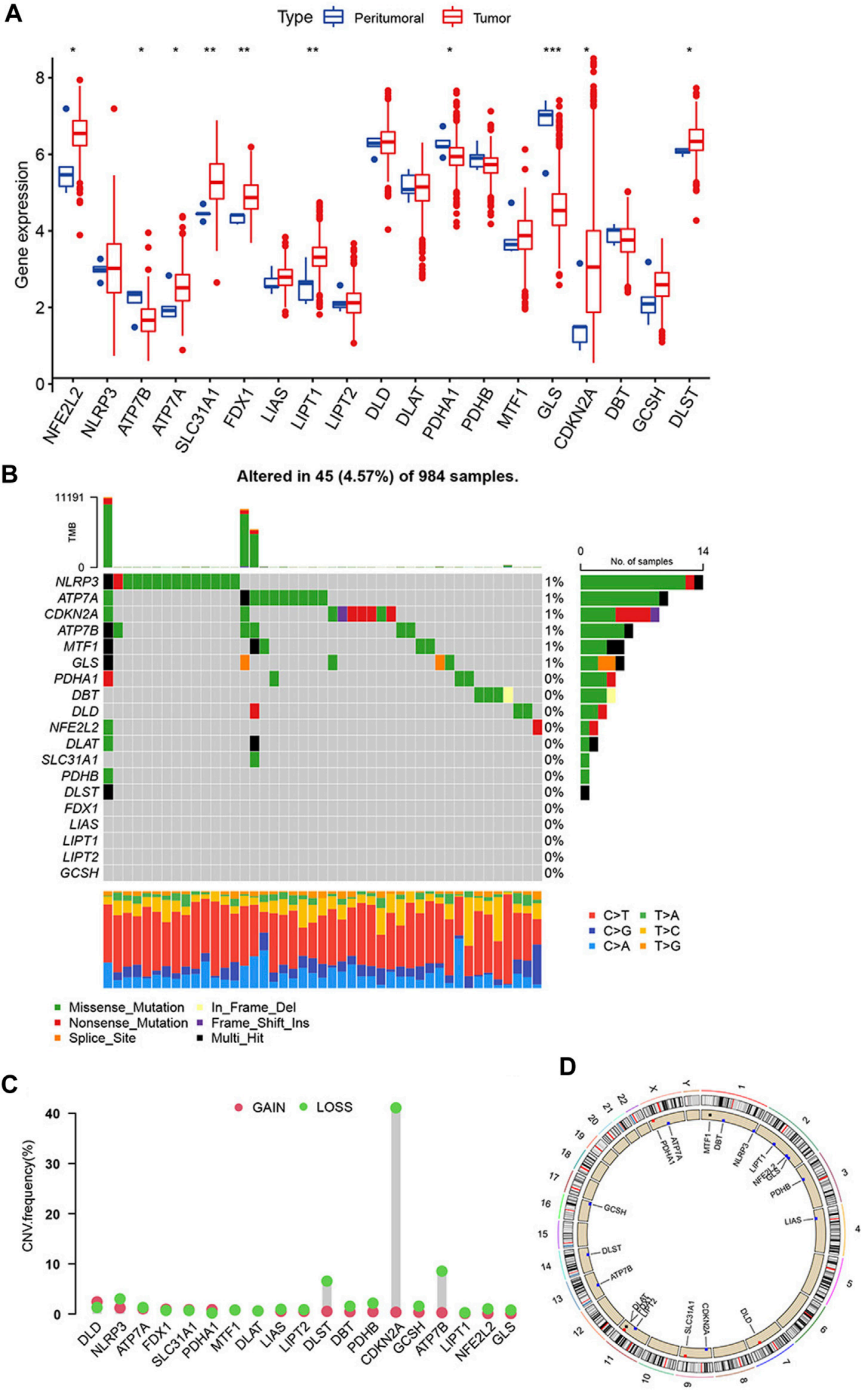
Genetic alterations of cuproptosis-related genes in glioma. **(A)** Expression of 19 cuproptosis-related genes in glioma and adjacent tissue. **(B)** Mutations of 19 cuproptosis-related genes in a cohort of 984 glioma patients’ samples. **(C)** CNVs frequencies of cuproptosis-related genes. **(D)** Location of cuproptosis-related genes on chromosomes and their CNV alterations. (*p < 0.05; **p < 0.01; ***p < 0.001).

19 cuproptosis-related genes were identified based on previous research. We analyzed the transcriptome profiling data of TCGA-GBM and TCGA-LGG cohorts to identify differences in mRNA expression between glioma and adjacent tissues. The expression of *GLS* (*p* < 0.001), *LIPT1*, *FDX1*, *SLC31A1* (*p* < 0.01), *DLST*, *CDKN2A*, *PDHA1*, *ATP7A*, *ATP7B*, and *NFE2L2* (*p* < 0.05) was different between glioma and adjacent tissues (Figure 1A). A summary analysis of simple nucleotide variations in the GBM and LGG cohorts showed that of the 984 samples, 45 (4.57%) had mutations in the cuproptosis-related genes. Only *NLPR3*, *ATP3A*, *CDKN2A*, *ATP3B*, *MTF1*, and *GLS* had an approximately 1% mutation frequency (Figure 1B).

Next, we analyzed copy number variations (CNV) and discovered that all 19 cuproptosis-related genes had CNVs. Among these genes, *CDKN2A*, *ATP7B*, and *DLST* had widespread CNV loss, especially CDKN2A, which had a CNV loss frequency of >40% (Figure 1C). The comparison of 19 cuproptosis-related genes CNV with all CNV was showed in Supplementary Table S2. Figure 1D shows location of 19 cuproptosis-related genes on the chromosomes and their CNV alterations. However, the CNV changes do not adequately explain differences in mRNA expression between the adjacent and glioma tissues. This suggests that not only CNVs affecting the expression of cuproptosis-related genes, but other factors, such as DNA methylation and transcription, may affect the mRNA expression of cuproptosis-related genes. There is an association between *CDKN2A* mutations and the development and recurrence of gliomas (Varn et al., 2022). It could be an important indicator for molecular diagnosis, such as *IDH* mutation or *MGMT* methylation.

### 3.2 Prognostic related cuproptosis-related genes and identification of cuproptosis subtypes in glioma

**FIGURE 2:**
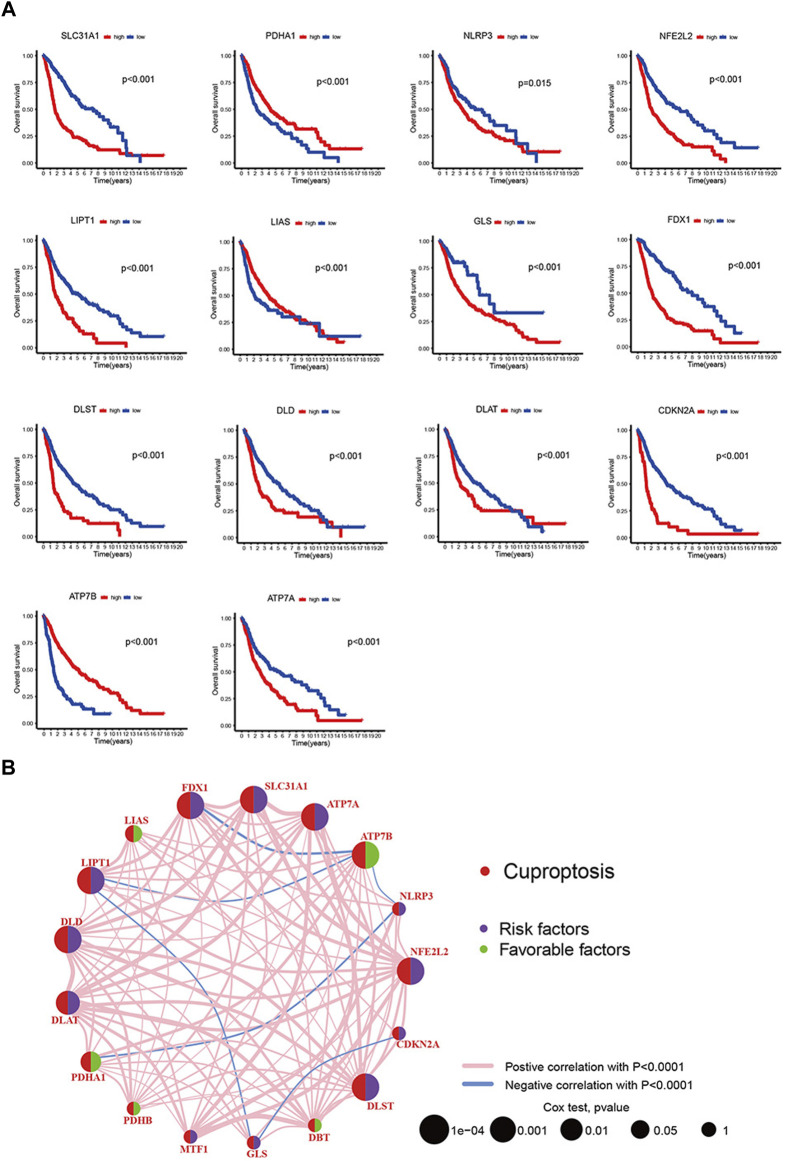
**(A)** 19 cuproptosis-related genes’ Kaplan-Meier analysis based on TCGA-LGG and TCGA-GBM cohorts. **(B)** Interactions, prognosis and regulator connections among cuproptosis-related genes in glioma.

We collected 3 eligible glioma cohorts (TCGA-LGG, TCGA-GBM, and GSE43378) to further study the expression patterns of cuproptosis-related genes that partake in tumorigenesis. A total of 725 patients were integrated from the 3 cohorts for further analysis (31 samples were removed because of the lack of complete clinical or gene expression information). Detailed patient information is presented in Supplementary Table S3. “Survival” and “survminer” packages were used to process the clinical data. The results of Kaplan-Meier analysis revealed the prognosis of cuproptosis-related genes, with *p* < 0.05. 14 of the 19 genes were thought to be associated with the prognosis of gliomas. Higher expression of *ATP7A, CDKN2A, DLAT, DLD, DLST, FDX1, GLS, LIPT1, NFE2L2, NLRP3*, and *SLC31A1* predicted poorer survival. *ATP7B, LIAS,* and *PDHA1* showed the opposite trend (Figure 2A). The Cox regression models of 19 cuproptosis-related genes were constructed to improve the survival analyses (Supplementary Table S4). The cuproptosis-related gene interactions, prognosis, and regulator connections are comprehensively illustrated in the cuproptosis network (Figure 2B).

**FIGURE 3:**
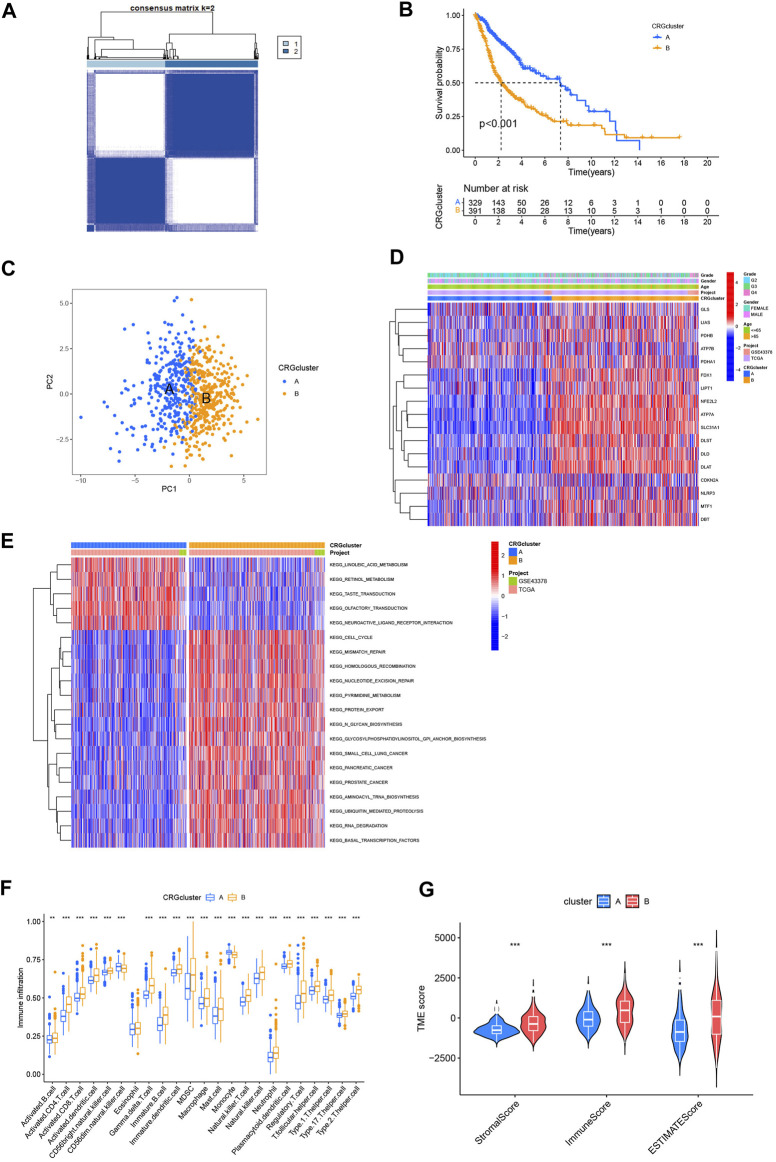
Analysis of cuproptosis subtypes. **(A)** Consensus clustering algorithm heatmap with k = 2. **(B)** Kaplan-Meier analysis of 2 subtypes. **(C)** PCA analysis. **(D)** Heatmap based on cuproptosis-related genes expression and pathology characteristics between 2 subtypes. **(E)** GSVA analysis of cuproptosis-related genes subtypes with red represent activated pathway and blue inhibited. **(F)** ssGSEA analysis of these 2 subtypes. **(G)** TME score of 2 subtypes. (*p < 0.05; **p < 0.01; ***p < 0.001.)

With the aim of examining the role of cuproptosis-related genes in glioma, a consensus clustering algorithm based on the PAM algorithm was used to categorize patients with glioma by analyzing their cuproptosis-related gene expression conditions. According to the algorithm results, it appears that when k = 2 the glioma cohort was optimally sorting into subtypes A and B (Figure 3A). The other consensus matrix, where k is valued from 3 to 9 were showed in Supplementary Figure S1. Subtype A included 333 patients, and subtype B included 392 patients. We used Kaplan-Meier analysis to compare the 2 subtypes, and the curves showed that compared to patients with subtype B, those with subtype A had a better survival probability (*p* < 0.001, Figure 3B). PCA analysis also revealed that there were differences between the 2 subtypes on cuproptosis-related gene expression profiles (Figure 3C). The heatmap showed that both cuproptosis-related gene expression and pathological characteristics are clearly different in the 2 subtypes (Figure 3D). More patients with grade 4 glioma were classified as subtype B, whereas more patients with grade 2 glioma were classified as subtype A. The heatmap also showed that most of cuproptosis-related genes were highly expressed in subtype B, while *LIAS, ATP7B, PDHA1*, and *CDKN2A* were expressed higher in subtype A. Also subtype B had more patients aged >65. These characteristics may explain the prognostic differences between the 2 cuproptosis subtypes.

GSVA analysis was used to explore the TME differences between the 2 cuproptosis subtypes (Figure 3E). Subtype A converged on lipid metabolism, particularly linoleic acid and retinol. Subtype B converged on pathways associated with the metabolism and damage repair of hereditary substances, including mismatch repair, homologous recombination, and nucleotide excision repair significantly. In addition, cell cycle and protein metabolism pathways are worthy of attention in subtype B. GSVA analysis revealed that the cell cycle and metabolism may cause different prognoses between the 2 cuproptosis subtypes.

In tumorigenesis and development of glioma, tumor immune microenvironment plays a critical part. Understanding how cuproptosis relates to the immune landscape in the tumor microenvironment may provide new avenues to treat cancer (Klemm et al., 2020). Therefore, we used ssGSEA to determine whether there was an immune infiltration difference between the cuproptosis subtypes (Figure 3F). A significant difference was found in the level of immune infiltration based on the analysis. Subtype B showed a higher infiltration level, especially in activated CD4^+^ and CD8^+^ T-cell, gamma delta T-cell, natural killer (NK) T-cell, and NK cells. We then used “estimate” package to compute the TME score of two subtypes (Figure 3G). In contrast to patients with Subtype A, those with Subtype B got higher stromal, immune, and ESTIMATE scores (p0.001). This suggests that the TME of patients with Subtype B has a higher proportion of stromal cells or immune cells.

The results showed an association between cuproptosis-related genes expression and glioma prognosis. We constructed 2 subtypes based on cuproptosis. The analysis revealed that the cell cycle, tumor metabolism, and immune infiltration may be related to cuproptosis in glioma.

### 3.3 Identification of gene subtypes based on differentially expressed genes in cuproptosis subtypes

**FIGURE 4:**
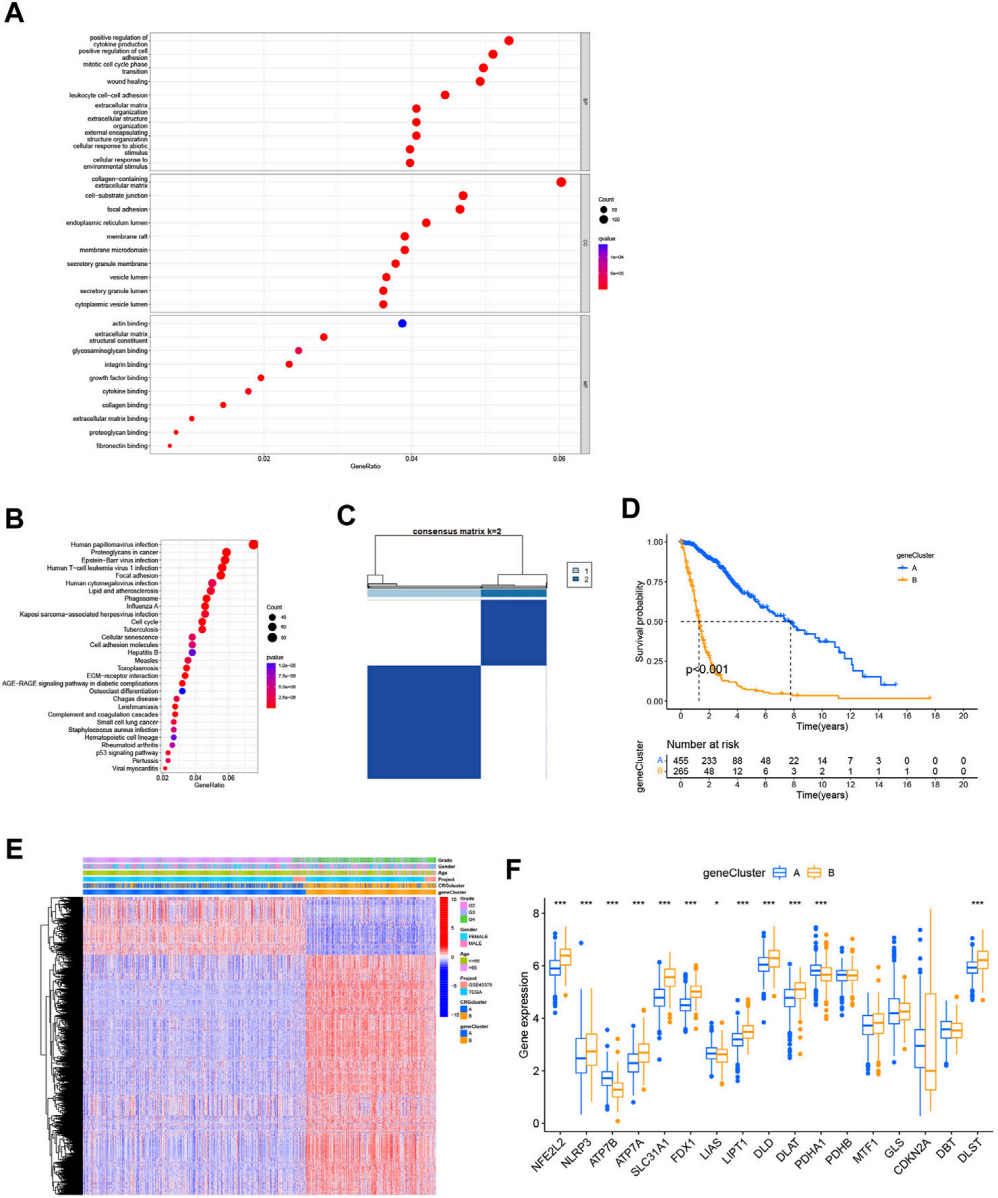
Identification of the gene subtypes. **(A,B)** KEGG and GO enrichment analysis of cuproptosis subtype related genes. **(C)** Consensus clustering algorithm heatmap with k = 2 decided on 2 gene subtypes. **(D)** Kaplan-Meier analysis of 2 gene subtypes. **(E)** Heatmap of gene subtypes. **(F)** Differences of cuproptosis-related genes expression between 2 gene subtypes. (*p < 0.05; **p < 0.01; ***p < 0.001).

We used the “limma” package to analyze differentially expressed genes in the 2 cuproptosis subtypes, with logFCfliter = 0.585 and adjusted *p*-value fliter = 0.05. We identified 2,486 differentially expressed genes, and we believe that these genes are related to potential biological differences between the cuproptosis subtypes. A volcano plot showed all differentially expressed genes and highlighted the cuproptosis-related genes (Supplementary Figure S2). Functional enrichment analysis was be conducted by a gene ontology database (Figure 4A). And those cuproptosis subtype-related genes were enriched for cytokine production, cell cycle, cell adhesion, and extracellular environment. An analysis of KEGG revealed the enrichment of proteoglycans in cancer, cell cycle, senescence, cell adhesion, and p53 signaling (Figure 4B). We could find that GO and KEGG are similar in the following ways: cell cycle, cell adhesion and extracellular environment (Figures 4A, B). We also believed that GO and KEGG confirmed GSVA results (Figure 3E), which shows different pathways between two cuproptosis subtypes such as KEGG_CELL_CYCLE, KEGG_PROTEIN_EXPORT and KEGG_MISMATCH_REPAIR. In addition, it was revealed that cuproptosis subtype-related genes may influence glioma cell characteristics and TME, and may contribute to glioma oncogenesis. To learn more about the significance of cuproptosis subtype-related genes in glioma prognosis, we used a consensus clustering algorithm based on the PAM algorithm. The 725 cohort patients were divided into 2 new subtypes: gene subtypes A and B with 459 and 266 patients, respectively (Figure 4C; Supplementary Figure S2). Kaplan-Meier analysis illustrated a significantly difference in prognosis between the 2 gene subtypes (*p* < 0.001, Figure 4D). The heatmap of gene subtypes showed that most elderly patients and patients with grade 4 glioma were classified as gene subtype B, explaining the prognostic differences in cuproptosis gene subtypes (Figure 4E). Significant differences were also found in cuproptosis-related gene expression between the 2 cuproptosis gene subtypes (Figure 4F).

### 3.4 Construction and validation of the prognostic CRG-score

The CRG-score was constructed using differentially expressed genes among the gene subtypes. Using "caret" package, we randomly distributed the cohort patients (4 patients were excluded due to incomplete survival and gene expression data) into training (n = 360) and test (n = 361) groups. Our next step was to select the appropriate prognostic genes related to the gene subtypes through LASSO analysis and multivariate Cox analysis. Following result of the former with the minimum partial likelihood deviance, we kept 34 genes (Supplementary Figures S3A, B). And result of the latter revealed 11 genes, including 2 low-risk genes (*NOG* and *MKX*) and 9 high-risk genes (*NBPF8, TSKU, AURKB, SLC25A43, P2RY6, STEAP1, CDK4, RARRES1,* and *KCNN4*). The CRG-score was established as follows using the multivariate Cox regression analysis (Supplementary Table S5). The coefficients were kept 4 decimal places.

$$\begin{array}{c} CRG-score=\left(-0.2885\;*\;NOG\right)+\left(-0.2045\;*\;MKX\right)\\ +\left(0.2943\;*\;NBPF8\right)+\left(0.3126*\;TSKU\right)\\ +\left(0.1503\;*\;AURKB\right)+\left(0.2898\;*\;SLC25A43\right)\\ +\left(0.2731\;*\;P2RY6\right)+\left(0.1496\;*\;STEAP1\right)\\ +\left(0.1252\;*\;CDK4\right)+\left(0.1361\;*\;RARRES1\right)\\ +\left(0.1671\;*\;KCNN4\right) \end{array}$$

**FIGURE 5:**
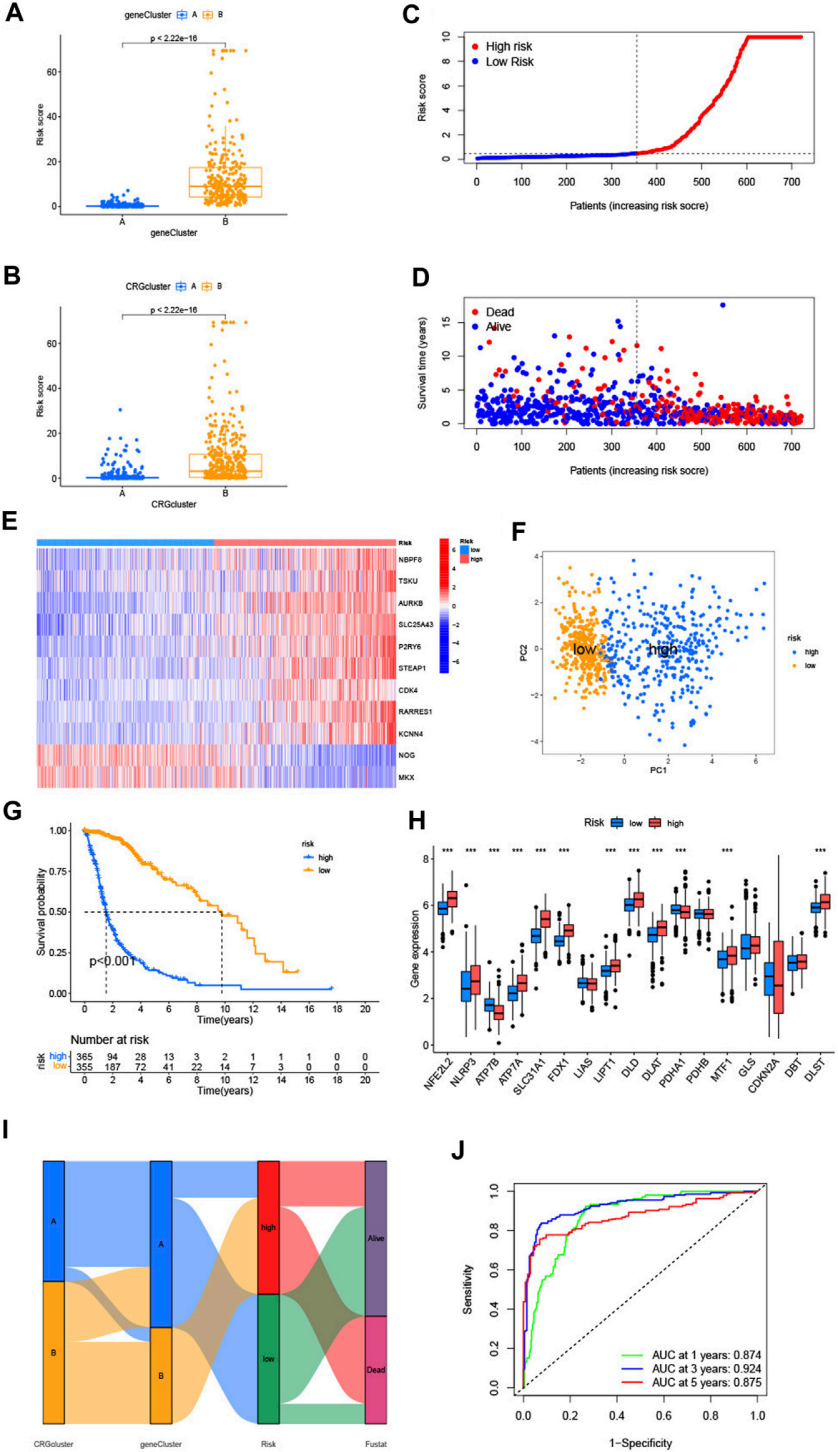
**(A,B)** Differences of CRG-score between 2 cuproptosis-related genes subtypes and gene subtypes. **(C,D)** Relationships between CRG-score and patients’ survival status. **(E)** The heatmap of the CRG-score related genes expressions and CRG-score. **(F)** The PCA analysis of low-risk and high-risk groups. **(G)** Kaplan-Meier analysis of low-risk and high-risk groups. **(H)** Expression of cuproptosis-related genes in low-risk and high-risk groups. **(I)** The entirety network of the subtypes, risk of CRG-score and survival status based on the corhorts. **(J)** The ROC curve showed 1-, 3-, and 5-year AUC values. (*p < 0.05; **p < 0.01; ***p < 0.001).

The CRG-score showed a significant difference between gene subtypes (Figure 5A). Patients with subtype A generally had a lower CRG-score, which means that these patients had a lower risk and better prognosis. Similar differences were found between the 2 cuproptosis subtypes (Figure 5B). This indicates that the CRG-score may have relevance to immune infiltration, metabolism, and cell cycle.

Compared with the median risk score, patients with a lower CRG-score were divided as the low-risk group (n = 356), and a higher score were divided as the high-risk group (n = 365) (Figure 5C). According to the distribution plot of the CRG-score risk, death rate from glioma rises while survival times decrease with mounting risk score (Figure 5D). The heatmap of the CRG-score-related gene expression in the 2 risk groups was also consistent with the formula of the CRG-score (Figure 5E). PCA analysis showed the dimensions of the low-risk and high-risk group (Figure 5F). Kaplan-Meier analysis indicated that patients in the high-risk group had a significantly poor prognosis (Figure 5G). The expression of cuproptosis-related genes varied widely, which confirmed the prospective connection between the CRG-score and cuproptosis (Figure 5H). Sankey diagram illustrates entire network of the subtypes, risk of CRG-score, and survival status (Figure 5I). The ROC curve represented the survival rate of the CRG-score with area under the curve (AUC) values of 0.874, 0.924, and 0.875, which contain respectively the 1-, 3-, and 5- year (Figure 5J).

To validate the prognostic performance of the CRG-score, we computed the CRG-score of an external validation group (GSE83300) and the test group which was randomly chosen from the cohort by “caret” packages. The distribution plot, heatmap, Kaplan-Meier analysis, and ROC curve analysis are shown in Supplementary Figures S5, S6. Consistent with the previous results, the low-risk group had better prognosis. The AUC values at the 1-, 3-, and 5-year were relatively high, which indicates that the CRG-score had a satisfactory capability to predict the survival of patients with glioma patients.

### 3.5 The validation of CRG-score related genes’ expression by clinical samples

We used RT-qPCR to analyze the expression of 11 CRG-score-related genes in gliomas and adjacent tissues (n = 5). The results are shown in Supplementary Figure S7.

### 3.6 Evaluation of immune correlation and TME between low- and high-risk groups

**FIGURE 6:**
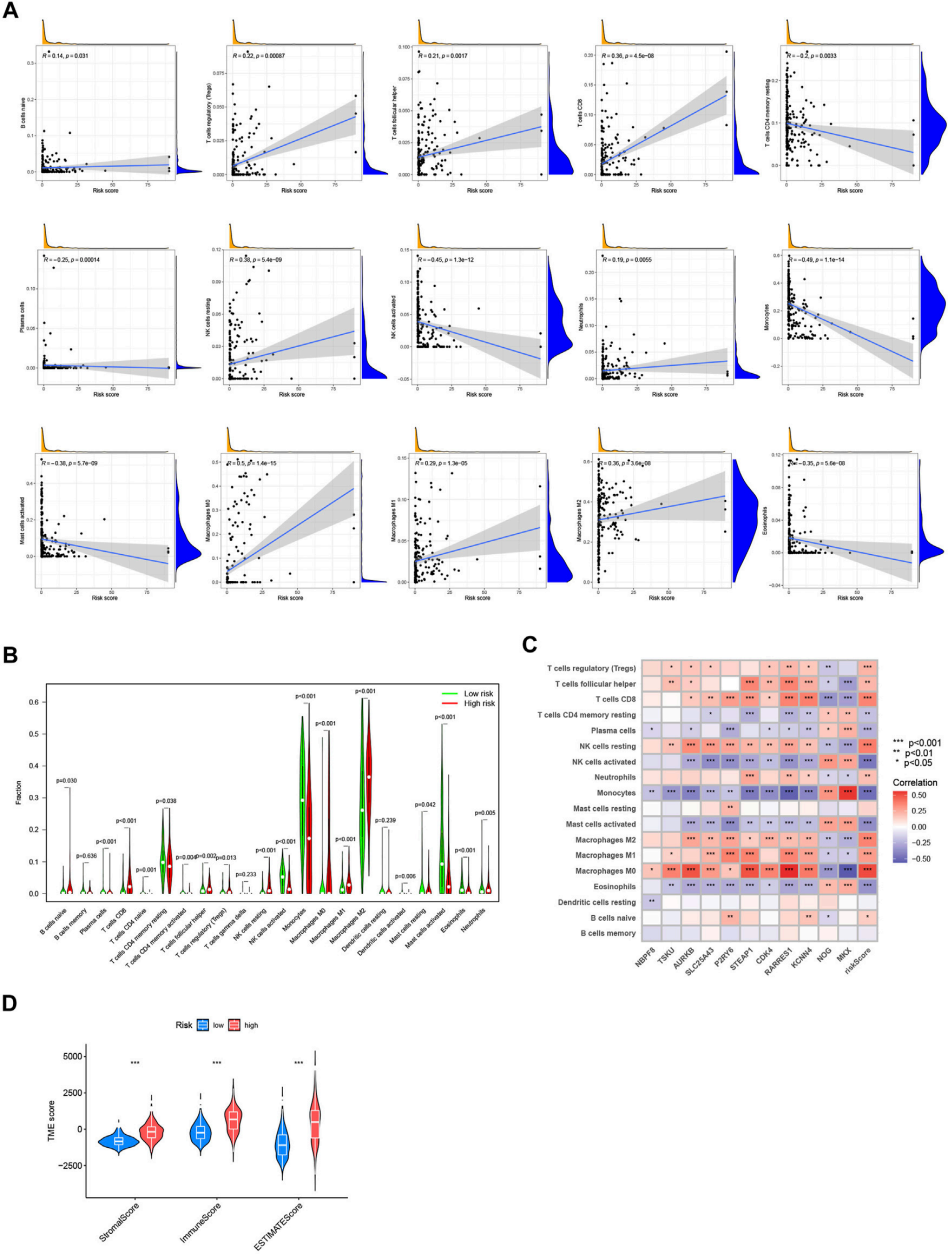
Evaluation of immune correlation and TME. **(A)** The connections between CRG-score and immune cells abundance. **(B)** Wilcoxon test of CRG-score and immune cells abundance. **(C)** The connections between the genes in CRG-score model and abundance of immune cells. **(D)** The TME score of 2 risk groups. (*p < 0.05; **p < 0.01; ***p < 0.001).

With the CIBERSORT algorithm, we further explored the potential role of immune infiltration by evaluating the association between the CRG-score and the abundance of immune cells. The CRG-score was positively correlated with Tregs, CD8^+^ T-cell, follicular helper T-cell, resting NK cells, neutrophils, M0, M1 and M2 macrophages. It presented negative correlation with resting memory CD4^+^ T-cell, plasma cells, activated NK cells, monocytes, activated mast cells, and eosinophils (Figure 6A). And a vioplot used Wilcoxon test was more significantly showed the relationship between immune infiltration and CRG-score (Figure 6B). Further, the CRG-score model genes were also correlated to the abundance of most immune cells significantly (Figure 6C). Using “estimate” package, we evaluated the TME score of low- and high-risk group (Figure 6D). A high CRG-score was associated with a high stromal and immune score, which indicated a close relationship between the CRG-score and TME.

### 3.7 Mutation and cancer stem cell analysis

**FIGURE 7:**
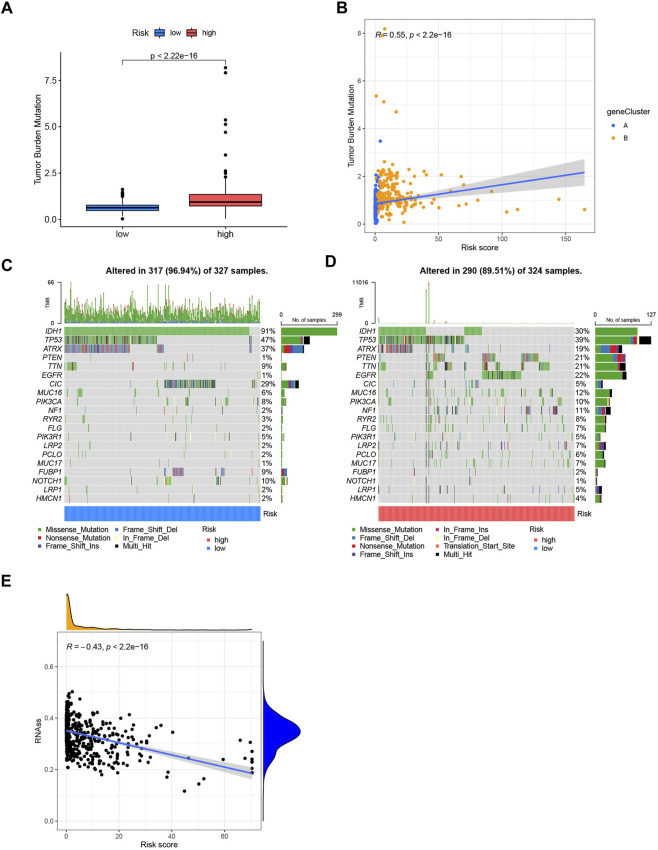
**(A)** Tumor mutation burden of low- and high-risk groups. **(B)** Spearman correlation analysis of CRG-score and tumor mutation burden. **(C,D)** Somatic mutations differences of the low and high-risk groups. **(E)** The correlation between the CRG-score and the cancer stem cell index.

The mutation data of the TCGA-LGG and GBM cohorts revealed a higher TMB index in the high-risk group (Figure 7A). Spearman correlation analysis indicated that the CRG-score was positively correlated with TMB (Figure 7B). We analyzed the somatic mutations in low- and high-risk group (Figures 7C, D). Differences in classical genes related to oncogenesis and development in gliomas, such as *IDH1, TP53, ATRX, PTEN, EGFR, CIC,* and *PIK3CA*, were observed. The low-risk group had higher mutation frequency of *IDH1, TP53, ATRX, and CIC*, in contrast to the high-risk group that had higher mutation frequency of *PTEN, EGFR, MUC16,* and *PIK3CA*. The somatic mutation results were consistent with the existing researches about prognosis of the gliomas (Rasheed et al., 1997; Cancer Genome Atlas Research et al., 2015; Chen et al., 2019; Bjorkblom et al., 2022; Ferrer, 2023). We also found that there was negative correlation between the CRG-score and the cancer stem cell index, indicating that gliomas with higher CRG-score had less stem cell characteristics and a higher degree of cell differentiation (Figure 7E).

### 3.8 Construction of a nomogram to predict survival of glioma patients

**FIGURE 8:**
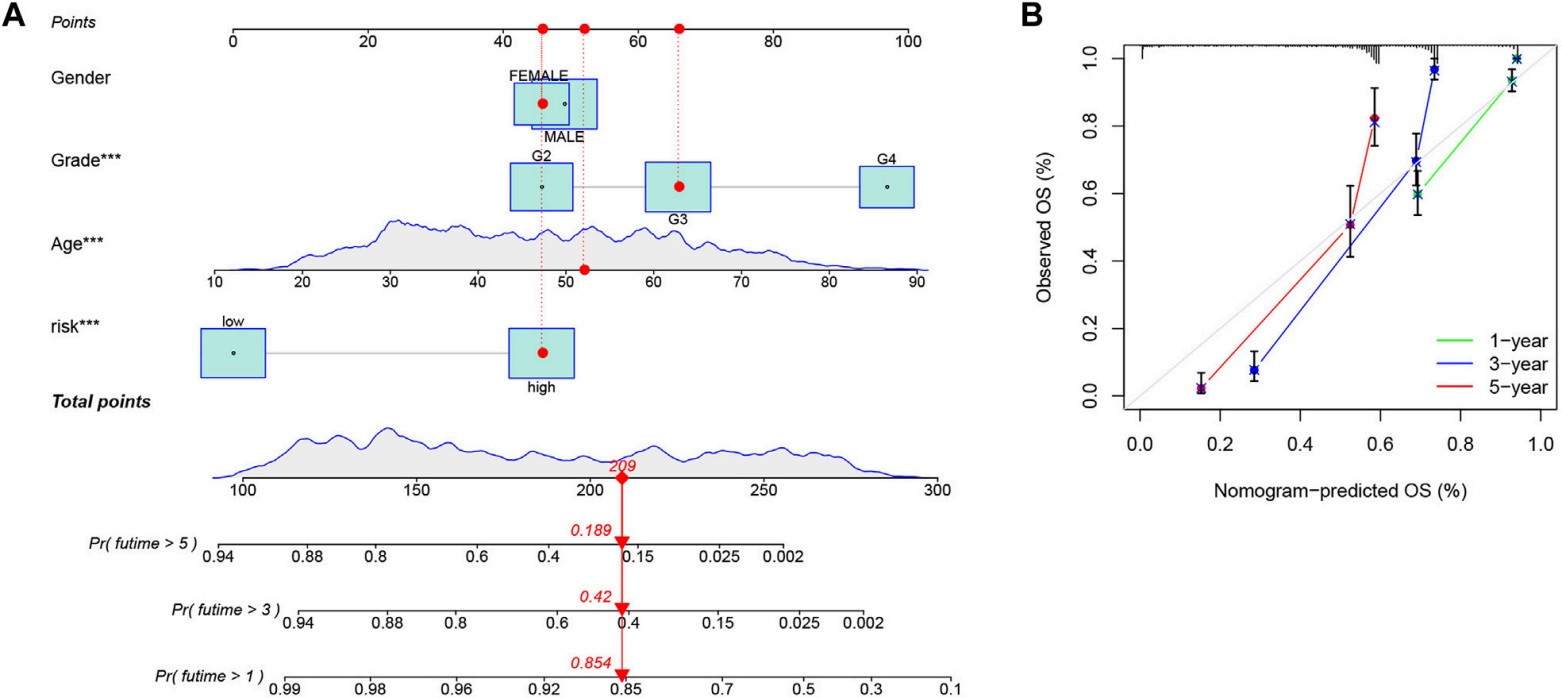
Construction of a nomogram. **(A)** Prediction of 1-, 3-, and 5-year survival, based on the CRG-score and clinicopathological characteristics by a nomogram. The corresponding values of Gender, Grade, Age and Risk group were obtained by using their positions at the “Points” abscissa. A patient’s total point is the sum of the four values. And by using the total points coordinate scale, we can get the prognosis prediction of the patient. **(B)** The calibration curve of the nomogram. The more the prediction curve matches the grey curve, the better the prediction effect will be proved.

Based on the CRG-score and clinicopathological characteristics, 1-, 3-, and 5-year survival could be predicted by a nomogram, which was constructed by “rms” package (Figure 8A). The calibration curve suggested that the nomogram was competent enough (Figure 8B).

### 3.9 Validations by clinical samples

**FIGURE 9:**
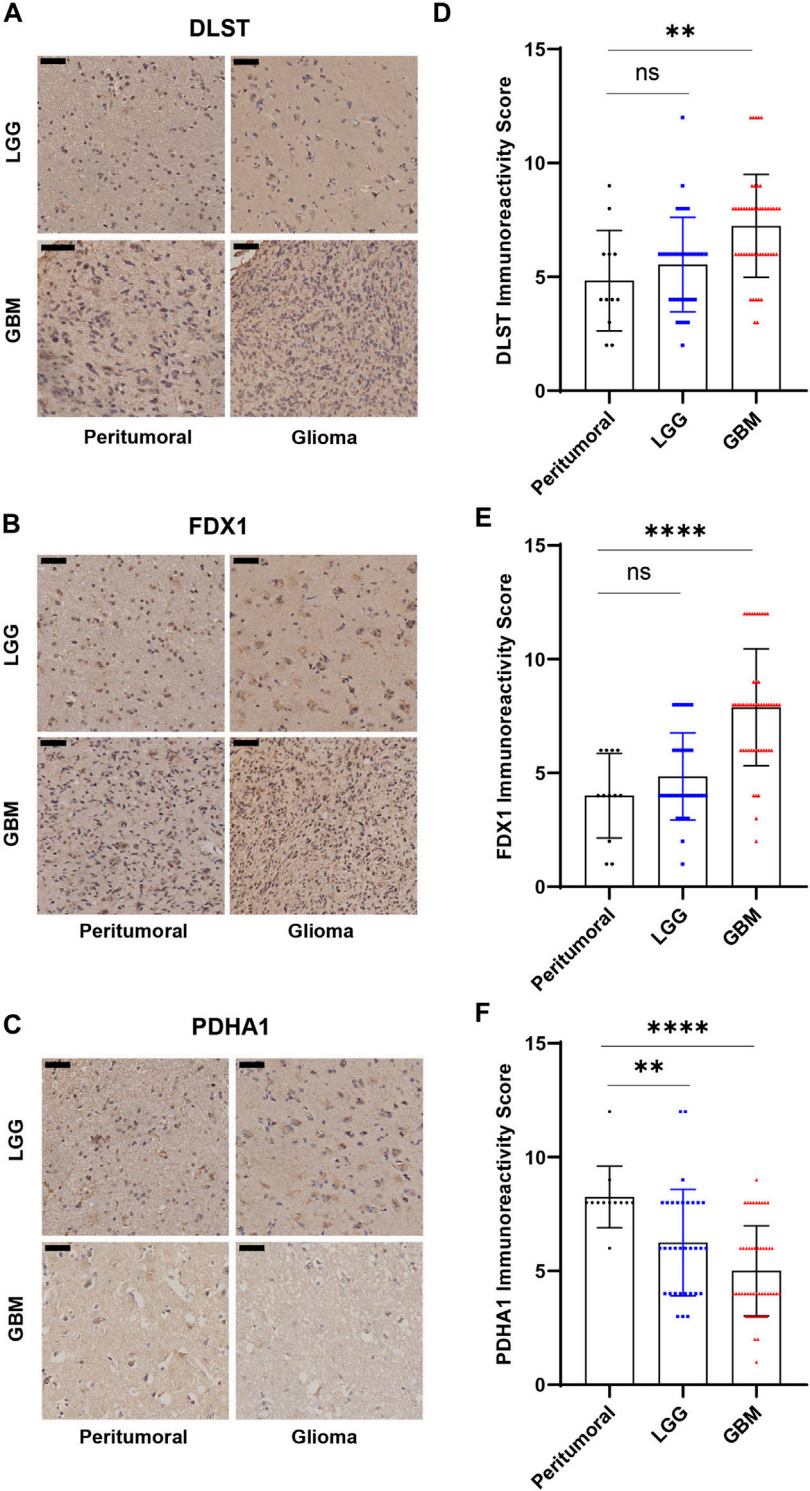
Immunohistochemical results of tissue microarrays. **(A–C)** Immunohistochemistry of DLST, FDX1, PDHA1. **(D–F)** Statistical analysis of immunohistochemical grades. (*p < 0.05; **p < 0.01; ***p < 0.001).

83 glioma tissue samples, including 27 grade 2 samples, 6 grade 3 samples, 50 grade 4 samples, and 12 adjacent tissues, were made into tissue microarrays. FDX1, DLST and PDHA1 were chose for immunohistochemistry. FDX1 and lipoylation act as key regulators in cuproptosis. DLST is one of the enzymes where lipoylation occur. PDHA1 was one of the protein targets of lipoylation. So, we believed that these three indicators are representative in cuproptosis. The expression of FDX1, DLST, and PDHA1 was detected and graded (Supplementary Figure S8). The results showed a general difference between LGG, GBM, and adjacent tissues, which is consistent with the database analysis (Figure 9).

## 4 Discussion

Glioma is the most common malignant intracranial tumor, and has a poor prognosis. Most patients with GBM succumb to the disease within a year while approximately 5% have a 5-year survival rate (Ostrom et al., 2014). A large number of grade 2 and grade 3 glioma patients experienced tumor recurrence and increased tumor grade after surgery and radiochemotherapy. Targeted therapies, particularly genotype-targeted therapies are minimally effective (Chen et al., 2017; Nicholson and Fine, 2021). With increasing cell death research, multiple cell death models have been associated with tumor occurrence and development (Mou et al., 2019; Hou et al., 2020). A close relationship between cuproptosis and tumor development has been demonstrated since its discovery (Kahlson and Dixon, 2022; Tsvetkov et al., 2022). Because the combined effects of glioma pathogenesis and development by multiple cuproptosis-related genes have not been elaborated, we collected and comprehensively analyzed 19 cuproptosis-related genes to reveal the potential association between cuproptosis and glioma. We found changes at the genetic and transcriptomic levels of cuproptosis-related genes in LGG and GBM. Immunohistochemistry of FDX1, DLST, and PDHA1, which included 95 clinical tissues, verified genetic differences at the protein level. Based on this discrepancy, we identified two cuproptosis molecular subtypes in a cohort of 725 patients. Multiple analyses revealed notable differences between the 2 subtypes. In contrast with patients with subtype A, those with subtypes B had a lower survival probability. PCA also supported the differences between subtypes A and B. GSVA, KEGG, and GO analyses revealed that the 2 subtypes had differences in cell cycle, metabolism, immune infiltration, and other TME characteristics, which revealed the role of cuproptosis in glioma. We further investigated 2 gene subtypes identified by differentially expressed genes in cuproptosis subtypes. By analyzing the gene subtypes, we constructed a CRG-score to predict patient prognosis. We used the CRG-score to grade the patients in the cohort and divided them into a low- and high-risk group. The low- and high-risk group showed significant differences in prognosis, cuproptosis-related gene expression, infiltrated immunocytes, mutations, and other TME characteristics. To make the CRG-score more accurate and operable, we constructed a nomogram additionally. This cuproptosis-related model could explain the molecular mechanism of glioma to a certain extent and provide potential cuproptosis-related therapies for glioma.

Different from other types of tumor samples, in neurosurgery, we will try to remove tumors and avoid damaging any normal brain tissue. Collecting unnecessary normal brain tissue is ethical transgression. Therefore, in most researches related to glioma, adjacent tissues were used to replace normal brain tissues. In this study, clinical samples of patients we used were all adjacent tissues or glioma tissues needed to be surgically removed. And in TCGA databases, the naming of samples follows a principle, which a “-11A” tag represents normal tissue sample in all kinds of tumors. We could not determine whether the samples in TCGA-LGG and GBM database is normal or adjacent tissues. Although adjacent tissues were unlikely to be completely unaffected cells and signaling molecules from either immune response or the tumor itself, the glioma researches using TCGA databases and clinical samples nearly all used the same methods. So, we believed it did not affect our analyses.

In our study, we also found that cuproptosis cluster B overlapped heavily with the HGG and older patients could be framed as a confounder of the analysis. In order to verify whether age affected the cuproptosis subtypes. We counted the number of the elderly patients (>65 years old) and non-elderly patients in each glioma grade. We also counted their cuproptosis subtypes. We used chi-square test to verify whether age was relevant with cuproptosis subtypes in each glioma grade. And it showed no significant difference in all three grades (grade 2: *p* = 0.952; grade 3: *p* = 0.5626; grade 4: *p* = 0.7498). The age of the patients did not affect the cuproptosis subtypes in each glioma grades. We believed that TCGA-GBM database collected more elderly patients than non-elderly patients which led to a bias. The cluster B had more GBM patients which reflected a false image of more elderly patients.

*CDKN2A* is considered a negative regulator of apoptosis (Tsvetkov et al., 2022). *CDKN2A* is located at 9p21, which is also known as multiple tumor suppressor l (*MTS1*) or *p16* ^*INKa*^. *CDKN2A* binds to and inactivates the CDK4 complex, leading to cell cycle arrest (Liggett and Sidransky, 1998; Monzon et al., 1998). As a tumor suppressor, *CDKN2A* can affect tumor development when silenced by deletion, methylation, or other mechanisms (Clurman and Groudine, 1998; Liggett and Sidransky, 1998). Many intracranial tumors are associated with changes in *CDKN2A* (Ichimura et al., 1996; Bostrom et al., 2001; Sievers et al., 2020). Multiple research teams have analyzed *CDKN2A* as an independent predictor of poor survival in both LGG and GBM (Lassaletta et al., 2017; Aoki et al., 2018; Shirahata et al., 2018; Appay et al., 2019). In the 2021 WHO classification of CNS tumors, *CDKN2A/B* homozygous deletion became one of the bases of glioma classification, which emphasized the importance of *CDKN2A* in the development of glioma (Louis et al., 2021; Komori, 2022). In this study, we observed differences in *CDKN2A* expression. We also discovered that cuproptosis affects cell cycle-related pathways in glioma, indicating that it may be involved in glioma development. These findings confirmed a potential connection between cuproptosis and glioma, which offers novel explanation in the progression of glioma.

Glioma development is often accompanied by immune-cell infiltration. Due to the lack of better treatments for glioma, studies on the immune microenvironment of glioma are gradually increasing (Lim et al., 2018; Xun et al., 2021; Li et al., 2022). Macrophages and monocytes are associated with glioma prognosis (Pyonteck et al., 2013; Ochocka et al., 2021). In the microenvironment of gliomas, monocytes may arrive as antitumor cells and differentiate into protumor macrophages (Ochocka et al., 2021). M0, M1 (antitumor), and M2 (protumor) subpopulations of macrophages play a vital role in glioma prognosis and immunotherapy (Komohara et al., 2008; Wei et al., 2020). Transformation between macrophage subpopulations can change glioma prognosis (Wang et al., 2022). Other immune cells, such as Tregs, CD8^+^ T-cell, and NK cells, also affect the biological history of glioma (Hussain et al., 2006). Activation of NK cells can enhance the effect of killing glioma cells (Lupo and Matosevic, 2020). Mast cells are believed to play an important role in angiogenesis and TME remodeling (Huang et al., 2008; Liu et al., 2011). The high level of mast cells may cause a better prognosis (Chen et al., 2022). The increases of neutrophils and M2 macrophages were also observed in a IDH-WT cohort, which means may be poor prognosis (Wang et al., 2017). In our study, we analyzed TME based on the CRG-score. The immune-cell infiltration status of the low- and high-risk group was consistent with current research results. Validating the prediction accuracy of the CRG-score and showing that cuproptosis was closely associated with glioma immune infiltration. Providing a new perspective on the association between cuproptosis and gliomas.

## 5 Conclusion

Our study revealed extensive regulatory mechanisms of cuproptosis-related genes in the cell cycle, TME, clinicopathological characteristics, and prognosis of glioma. Moreover, we constructed a prognostic CRG-score and nomogram system to predict the prognosis of patients with glioma. This suggests the potential importance of cuproptosis in the treatment of glioma and potentially providing novel glioma research approaches.

## Data Availability

The datasets presented in this study can be found in online repositories. The names of the repository/repositories and accession number(s) can be found in the article/Supplementary Material.
