# Supplementary material for: Identification of cuproptosis-related subtypes and the development of a prognostic model in glioma
Source: Front Genet. 2023 Mar 1;14:1124439. doi: 10.3389/fgene.2023.1124439 (PMC10014798; doi:10.3389/fgene.2023.1124439)
Supplement: Supplementary file 2 [file DataSheet1.DOCX]

Supplementary Material

Article Title

First Author*, Co-Author, Co-Author

*** Correspondence:** Corresponding Author: email@uni.edu

## Supplementary Figures


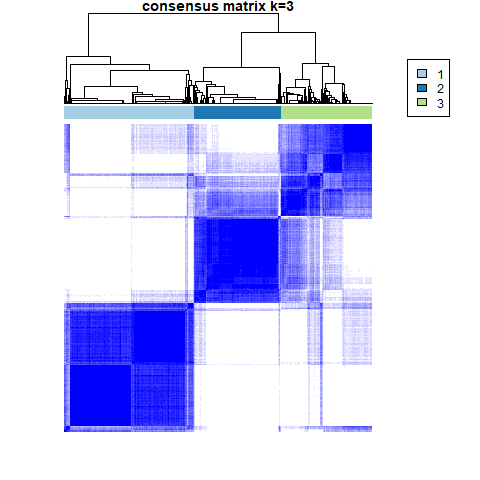

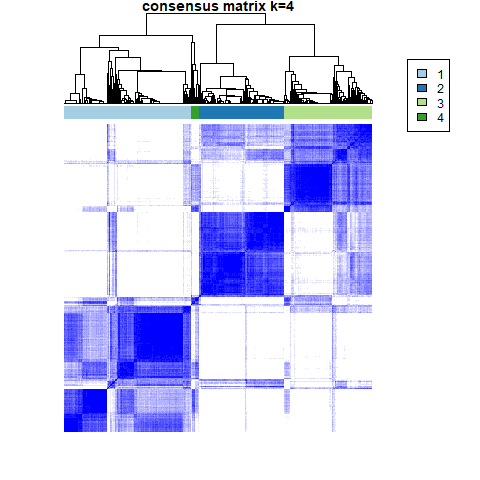

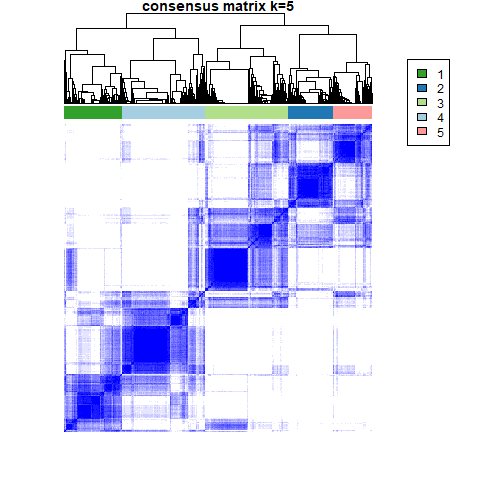

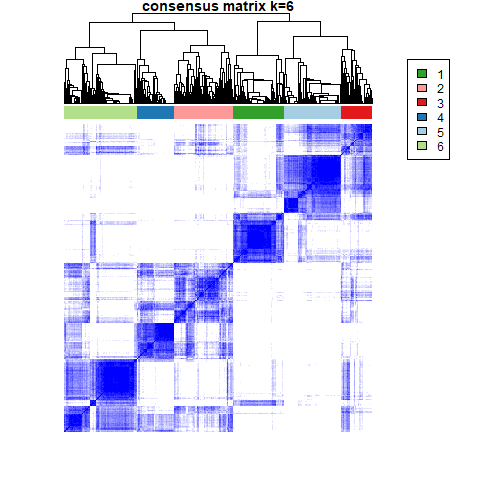

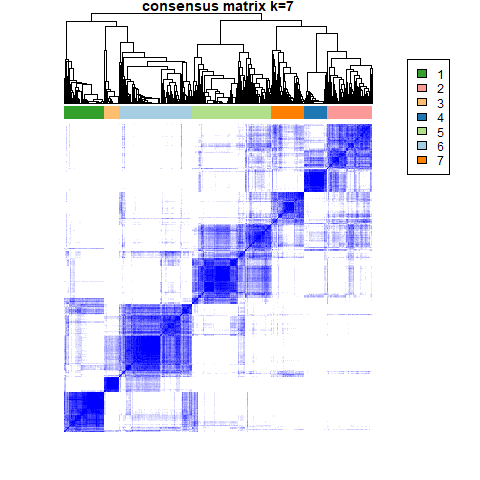

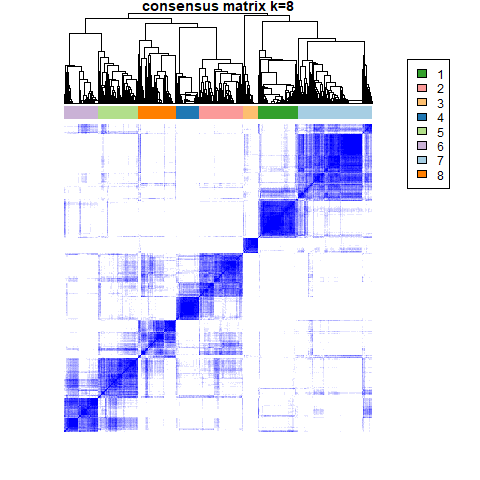

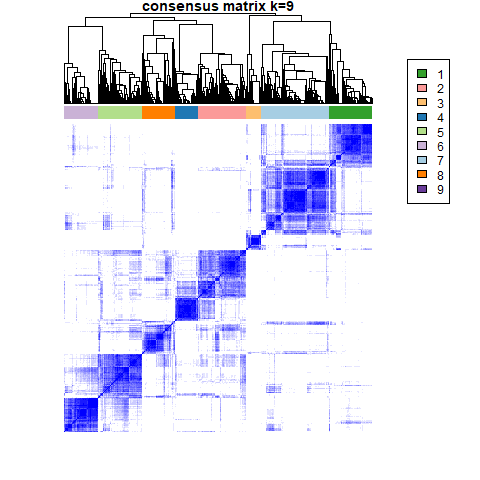


**Supplementary Figure 1**. Consensus matrix of cuproptosis subtypes, which k is valued from 3 to 9


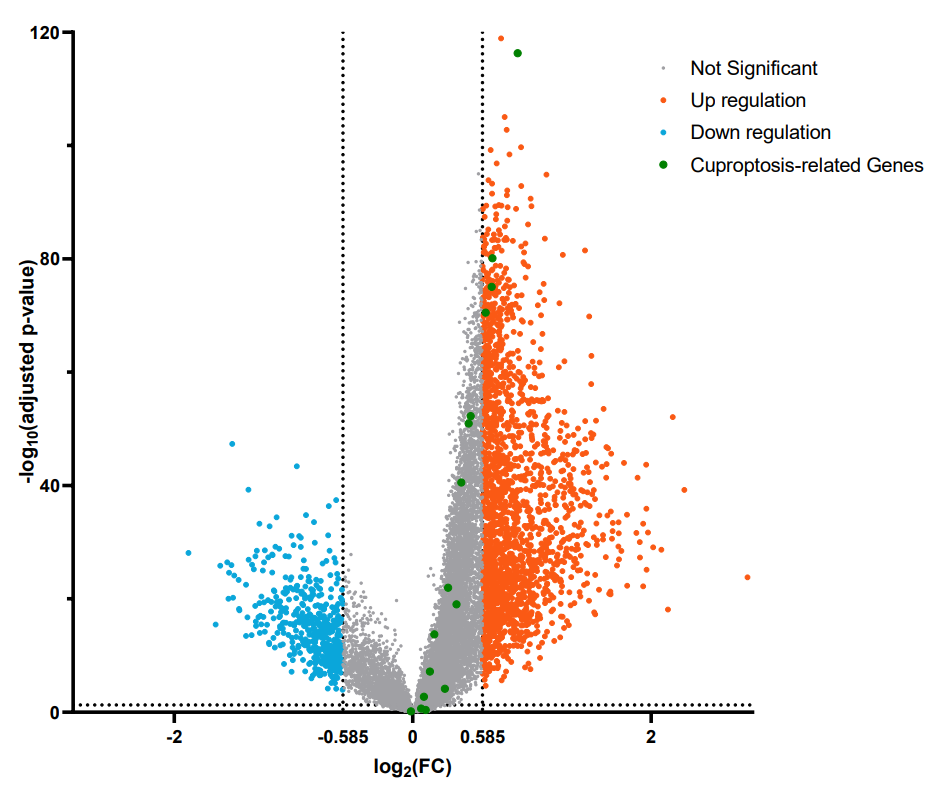


**Supplementary Figure 2.** Volcano plot of all differentially expressed genes and the cuproptosis-related genes


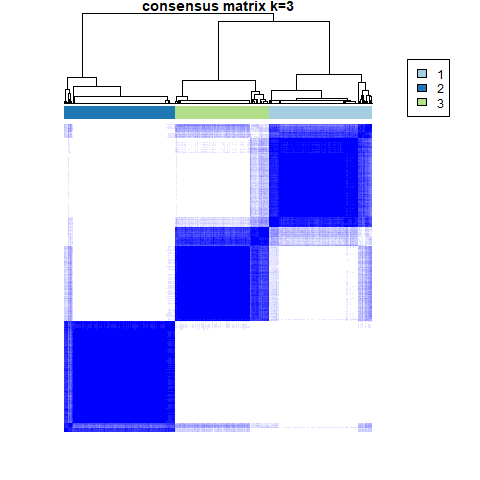

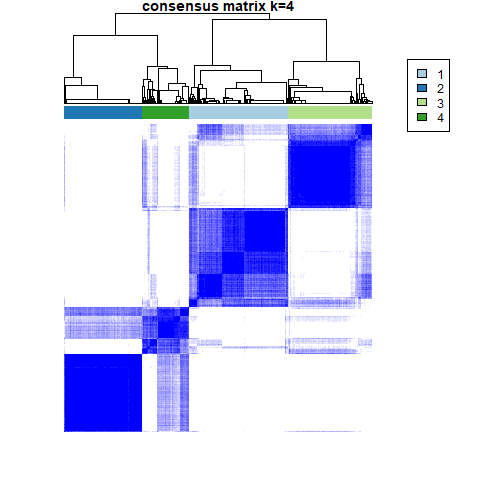

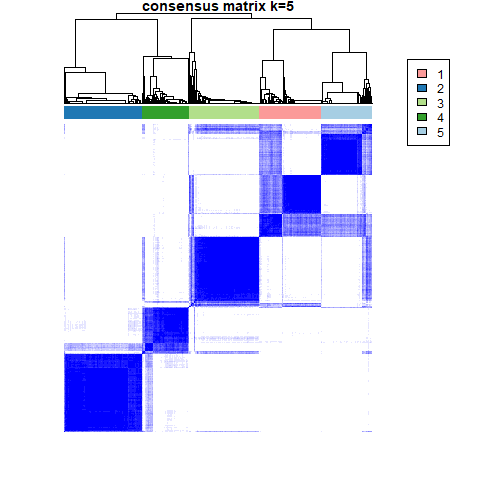

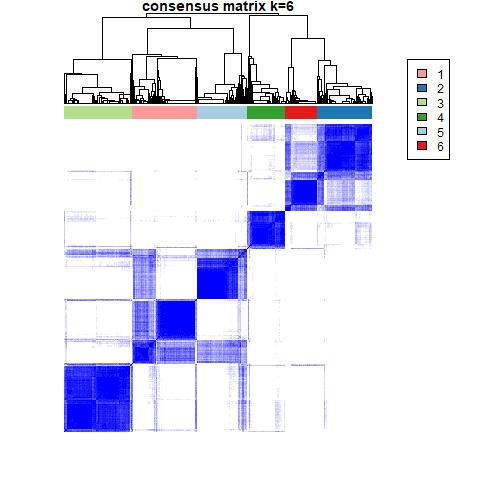

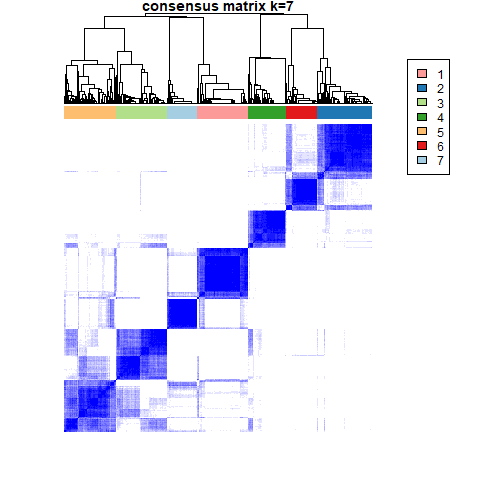

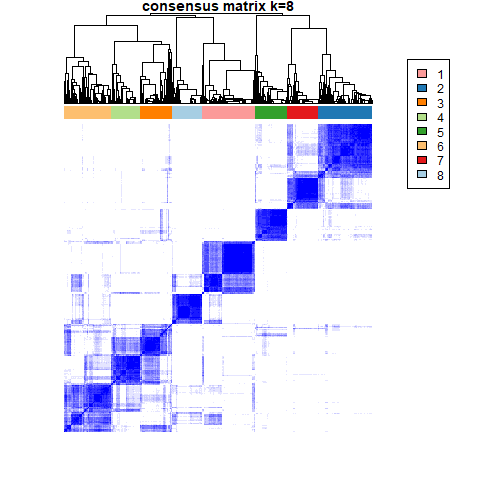

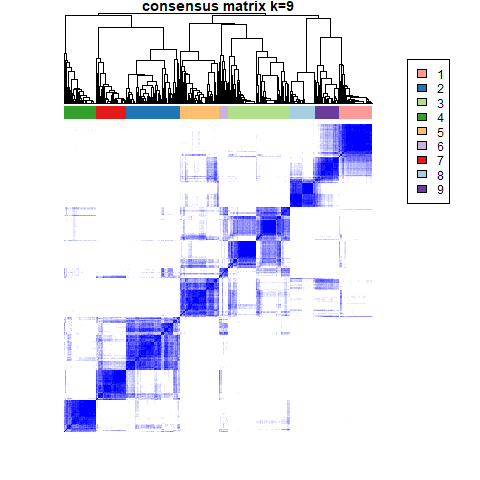


**Supplementary Figure 3.** Consensus matrix of cuproptosis gene subtypes, which k is valued from 3 to 9


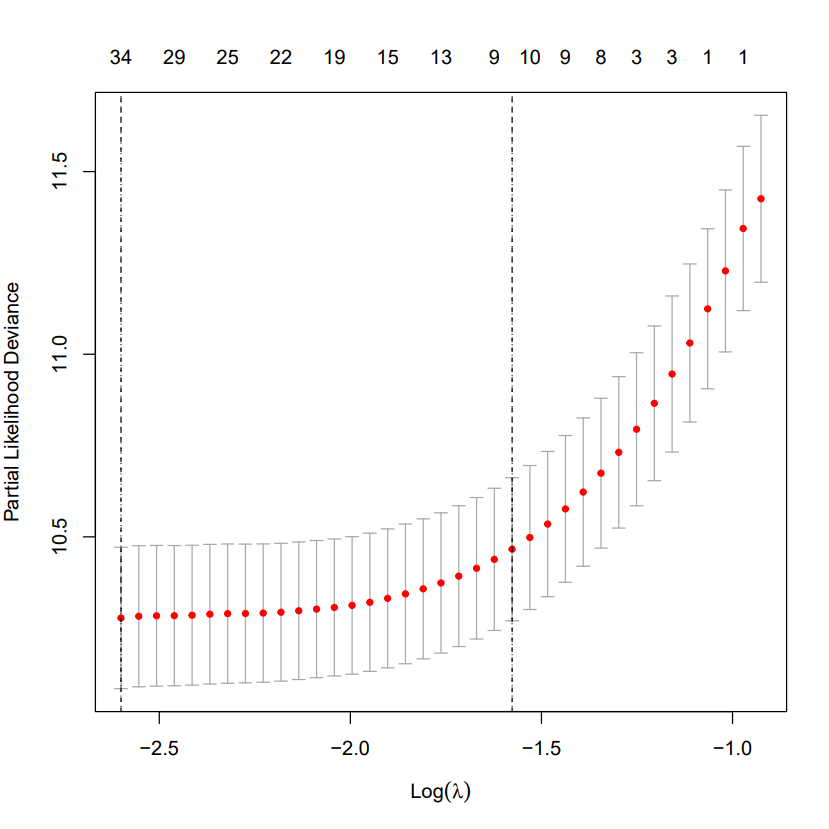

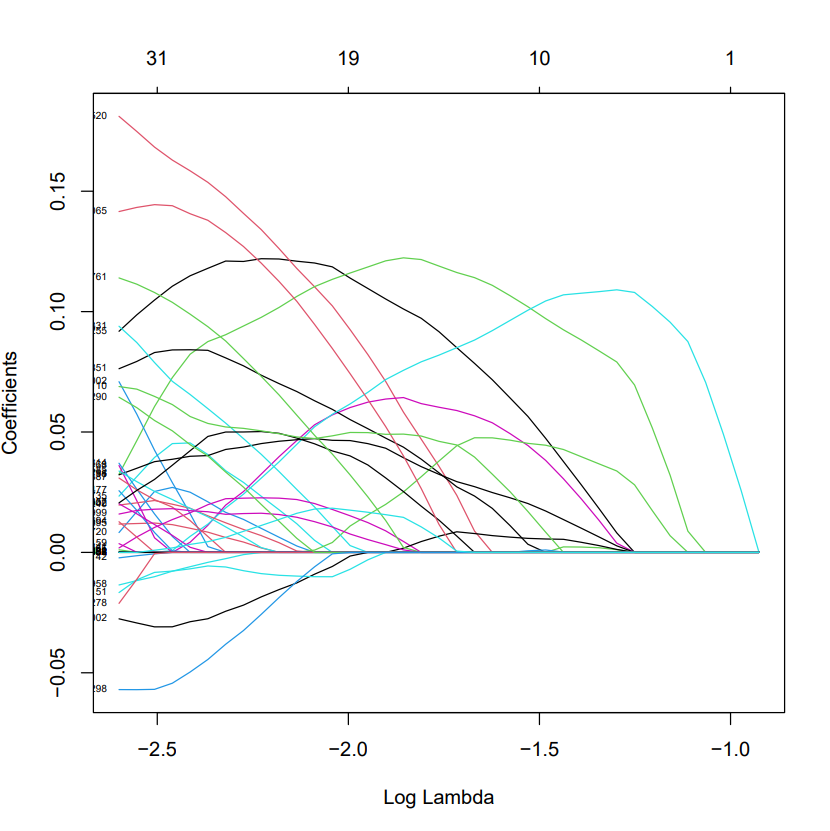


**Supplementary Figure 4.** LASSO analysis and multivariate Cox analysis


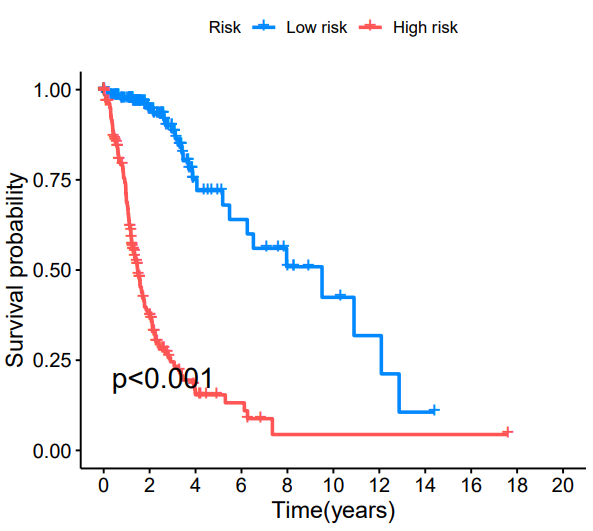

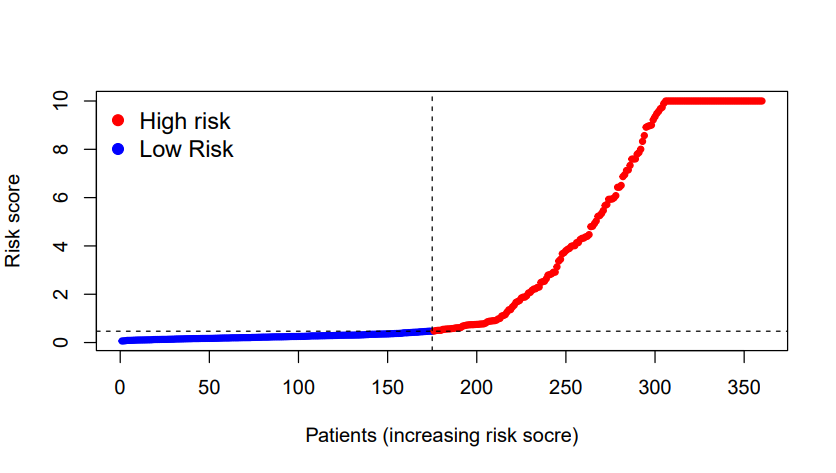

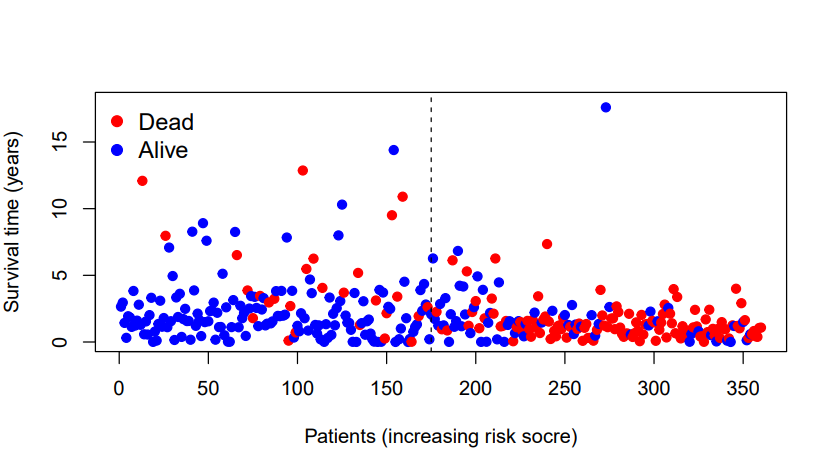

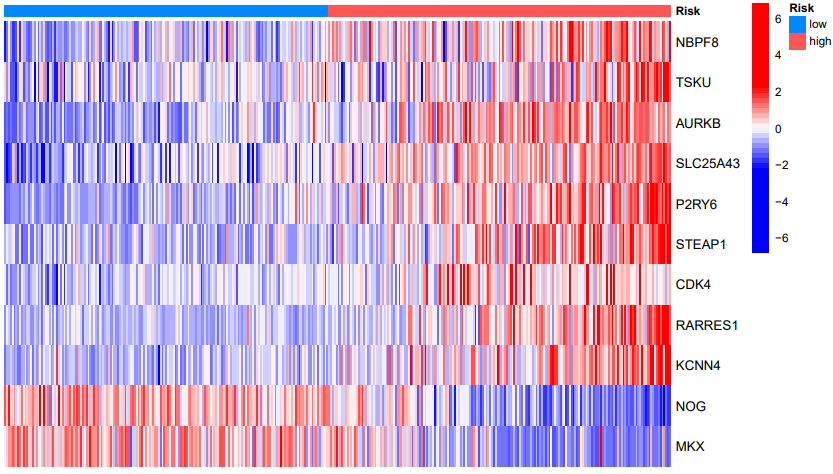

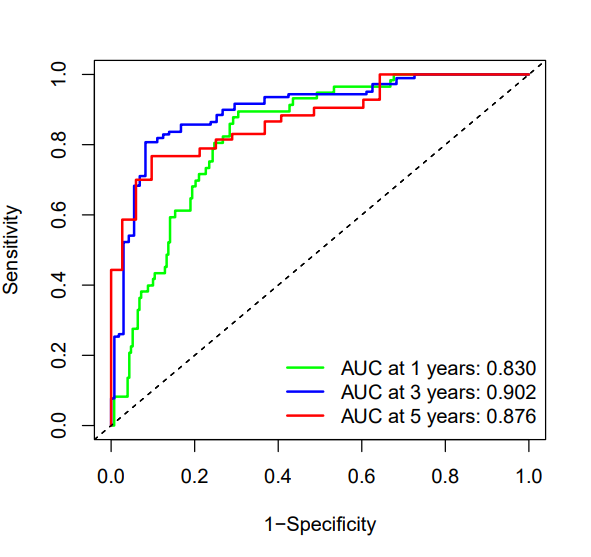


**Supplementary Figure 5.** The distribution plot, heatmap, Kaplan-Meier analysis, and ROC curve analysis of test group.


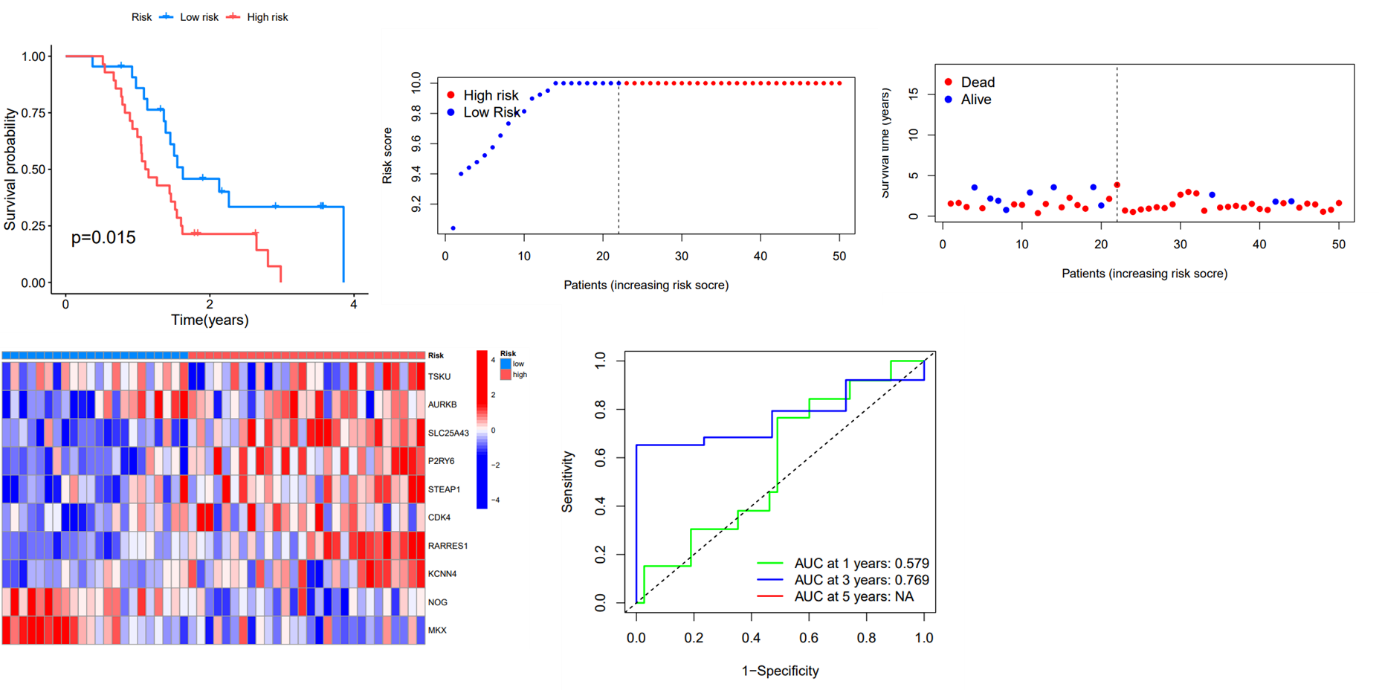


**Supplementary Figure 6.** The distribution plot, heatmap, Kaplan-Meier analysis, and ROC curve analysis of GSE83300 cohort.


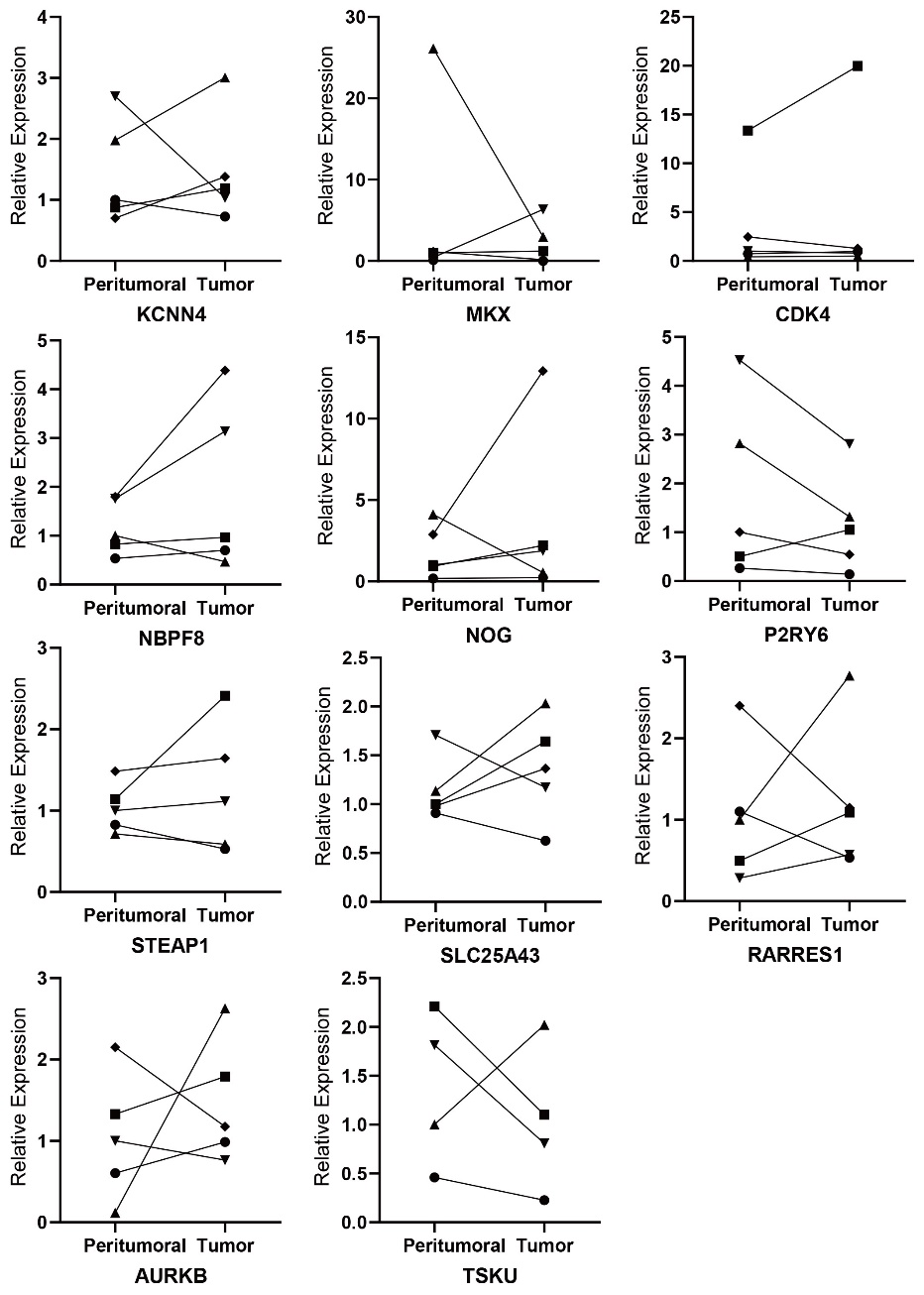


**Supplementary Figure 7.** RT-qPCR results of 11 CRG-score-related genes in glioma and their adjacent tissues (n=5).


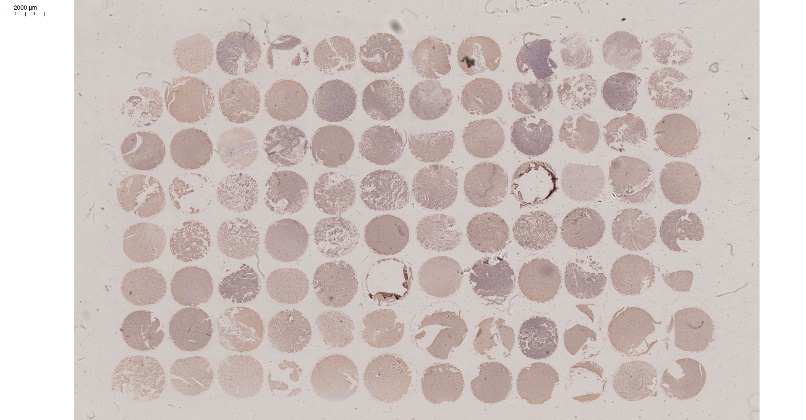

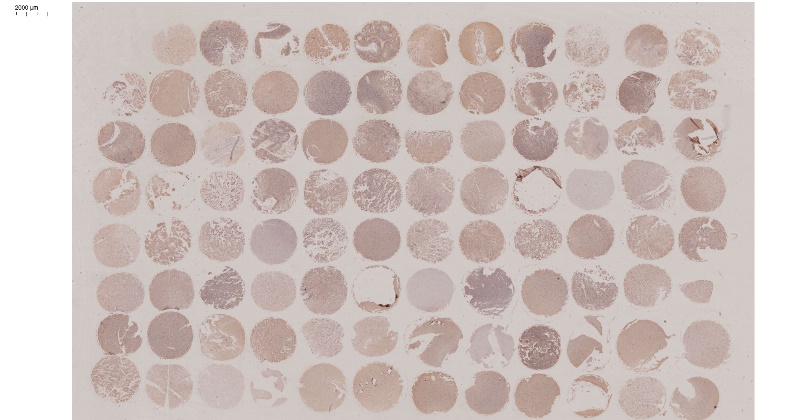

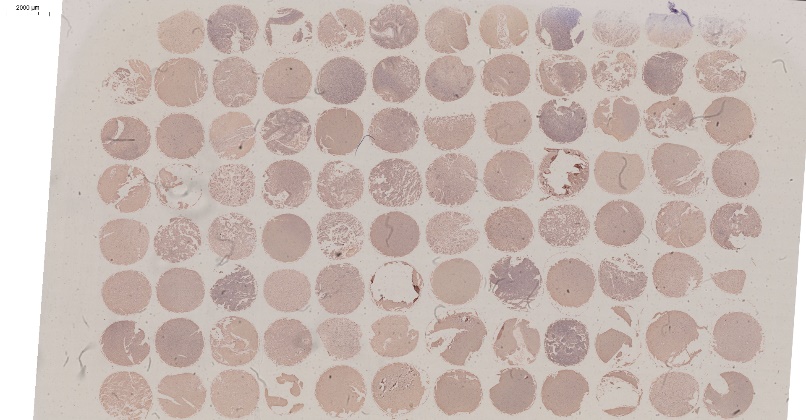


**A**

**B**

**C**

**Supplementary Figure 8.** The immunohistochemistry of tissue microarrays. **(A)** PDHA1. **(B)** FDX1. **(C)** DLST.
